# Supplementary material for: A Comprehensive Study of Meat Quality and Flavor Characteristics of Different Sexes of Yanbian Yellow Cattle Using GC-IMS and LC-MS/MS Technologies
Source: Foods. 2025 Sep 12;14(18):3175. doi: 10.3390/foods14183175 (PMC12468707; doi:10.3390/foods14183175)
Supplement: Supplementary file 1 [file foods-14-03175-s001.zip › Table S1.pdf]

Table S1. PEN-3 electronic nasal (E-nose) sensor-sensitive substances.

Array number	Sensor	Substances for sensing
R1	W1C	Aromatic hydrocarbons
R2	W5S	Nitrogen oxides
R3	W3C	Ammonia and other odorous components
R4	W6S	Hydrides
R5	W5C	Aromatic short-chain alkanes
R6	W1S	Alkanes
R7	W1W	Inorganic sulfides
R8	W2S	Alcohols, aldehydes and ketones
R9	W2W	Aromatic compounds, organosulfur compounds
R10	W3S	Long-chain alkanes
