# Supplementary material for: A Comprehensive Study of Meat Quality and Flavor Characteristics of Different Sexes of Yanbian Yellow Cattle Using GC-IMS and LC-MS/MS Technologies
Source: Foods. 2025 Sep 12;14(18):3175. doi: 10.3390/foods14183175 (PMC12468707; doi:10.3390/foods14183175)
Supplement: Supplementary file 1 [file foods-14-03175-s001.zip › Table S2.pdf]

Table S2. Amino acid content of Yanbian yellow cattle of different sexes.

Amino acid species	*Triceps brachii*		*longissimi dorsi*		*Gluteus medius*	
	Cow	Bull	Cow	Bull	Cow	Bull
Asp	1.46±0.11^{b*}	1.75±0.10^{b}	1.38±0.01^{c}	1.47±0.07^{c}	1.62±0.01^{a*}	1.95±0.00^{a}
Glu	2.46±0.07^{a*}	2.74±0.14^{b}	2.12±0.08^{b}	2.21±0.14^{c}	2.51±0.04^{a*}	3.16±0.01^{a}
UAA	3.92±0.04^{b*}	4.49±0.25^{b}	3.50±0.07^{c}	3.69±0.21^{c}	4.14±0.02^{a*}	5.12±0.01^{a}
※ Thr	0.78±0.04^{a}	0.84±0.03^{b}	0.66±0.02^{b}	0.68±0.04^{c}	0.75±0.05^{a*}	1.02±0.05^{ab}
Ser	0.90±0.00^{b*}	1.02±0.05^{b}	0.77±0.01^{c}	0.75±0.02^{c}	0.97±0.01^{a*}	1.15±0.00^{a}
Gly	1.34±0.06^{b*}	1.52±0.07^{a}	1.14±0.00^{c*}	1.42±0.10^{b}	1.49±0.09^{a}	1.62±0.01^{a}
Ala	1.61±0.01^{b*}	1.80±0.11^{b}	1.39±0.02^{c*}	1.61±0.12^{c*}	1.83±0.08^{a*}	2.01±0.00^{a}
Pro	0.55±0.02^{a*}	0.62±0.03^{b}	0.45±0.02^{c*}	0.56±0.04^{c}	0.60±0.01^{a*}	0.68±0.00^{a}
Total sweet amino acids, SAAs	5.19±0.02^{b*}	5.82±0.31^{b}	4.45±0.07^{b*}	5.03±0.34^{c}	5.66±0.11^{a*}	6.50±0.04^{a}
※ Val	0.47±0.00^{a*}	0.57±0.01^{b}	0.45±0.03^{a}	0.49±0.04^{c}	0.47±0.02^{a*}	0.73±0.07^{a}
※ Ile	0.35±0.00	0.44±0.00^{b}	0.35±0.03	0.37±0.03^{c}	0.32±0.05*	0.58±0.07^{a}
※ Leu	1.21±0.00^{a*}	1.39±0.06^{b}	1.09±0.02^{b}	1.16±0.08^{c}	1.24±0.05^{a*}	1.64±0.04^{a}
Tyr	0.40±0.00^{a*}	0.48±0.01^{b}	0.34±0.00^{b*}	0.40±0.02^{c}	0.40±0.02^{a*}	0.57±0.00^{a}
※ Phe	0.66±0.00^{b}	0.70±0.04^{b}	0.56±0.01^{c}	0.60±0.04^{c}	0.78±0.05^{a}	0.78±0.00^{a}
※ Lys	1.13±0.00^{*b}	1.37±0.03^{b}	1.07±0.06^{b*}	1.18±0.10^{c}	1.20±0.00^{a*}	1.50±0.03^{a}
※ His	0.57±0.00^{b*}	0.65±0.04^{b}	0.52±0.01^{b}	0.53±0.03^{c}	0.67±0.00^{a*}	0.74±0.01^{a}
Arg	0.85±0.00^{b*}	0.95±0.05^{b}	0.74±0.00^{c*}	0.80±0.05^{c}	0.93±0.01^{a*}	1.09±0.00^{a}
Total Bitter Amino Acids, BAAs	5.68±0.02^{b*}	6.58±0.28^{b}	5.15±0.11^{c*}	5.57±0.42^{c}	6.05±0.08^{a*}	7.67±0.24^{a}
Cys	0.06±0.00^{b*}	0.07±0.00^{a}	0.04±0.00^{c*}	0.05±0.00^{c}	0.05±0.00^{a*}	0.06±0.00^{b}
※Met	0.38±0.00^{b}	0.36±0.00^{b*}	0.31±0.02^{c*}	0.32±0.00^{c}	0.41±0.00^{a*}	0.44±0.01^{a}
TAA	15.25±0.09^{b*}	17.34±0.86^{b}	13.46±0.22^{c*}	14.66±0.99^{c}	16.33±0.03^{a*}	19.80±0.30^{a}
EAA	5.59±0.03^{b*}	6.35±0.25^{b}	5.04±0.11^{c}	5.36±0.40^{c}	5.87±0.13^{a*}	7.47±0.29^{a}
NEAA	9.66±0.11^{b*}	10.90±0.61^{b}	8.41±0.10^{c*}	9.31±0.59^{c}	10.40±0.13^{a*}	12.30±0.04^{a}
EAA/TAA(%)	36.66±0.03^{b}	36.62±0.29^{b}	37.44±0.10^{a}	36.59±0.40^{b}	35.95±0.40^{c*}	37.70±0.96^{a}
EAA/NEAA(%)	57.87±0.01^{b}	58.26±0.41^{b}	59.02±0.20^{a}	57.57±0.67^{b*}	56.44±0.02^{c*}	60.57±0.65^{a}
Arg	0.85±0.00^{b*}	0.95±0.05^{b}	0.74±0.00^{c*}	0.80±0.05^{c}	0.93±0.01^{a*}	1.09±0.00^{a}

Note: a-c represents significant differences between sites of the same sex ($P < 0.05$); * represents significant differences between sexes of the same site ($P < 0.05$).

※Represents essential amino acids
