# Supplementary figures and images for: A Comprehensive Study of Meat Quality and Flavor Characteristics of Different Sexes of Yanbian Yellow Cattle Using GC-IMS and LC-MS/MS Technologies
Source: Foods. 2025 Sep 12;14(18):3175. doi: 10.3390/foods14183175 (PMC12468707; doi:10.3390/foods14183175)

Figure S1. Information on fatty acid standards.

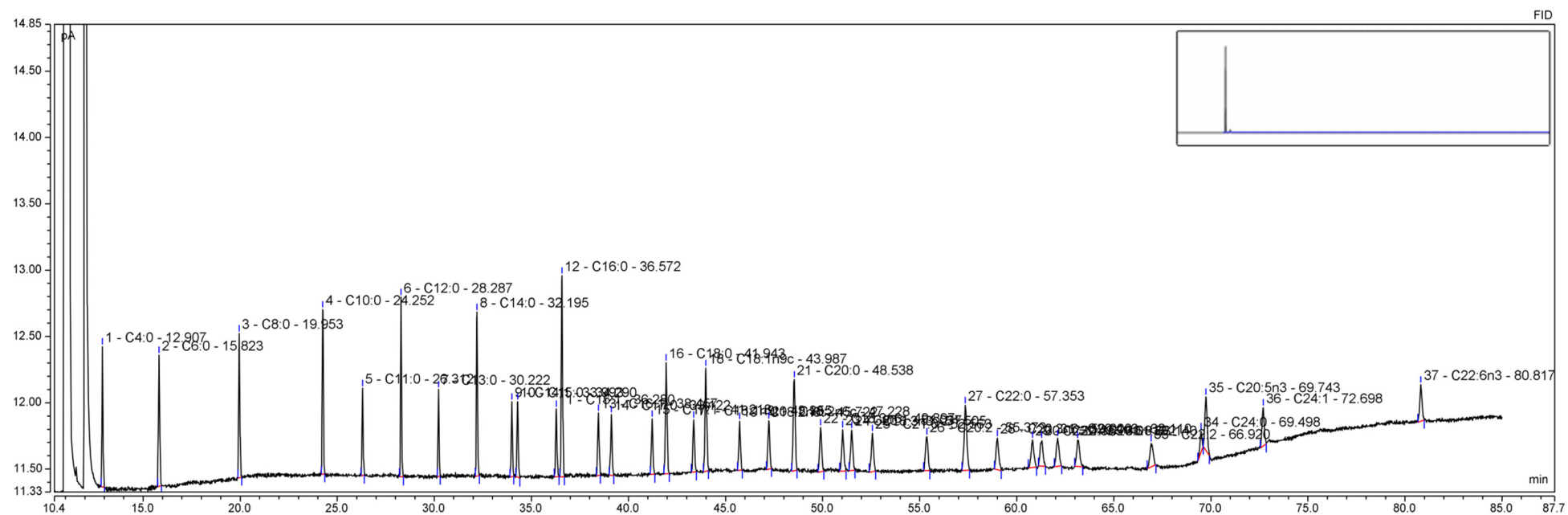

Supplement: Supplementary file 1 [file foods-14-03175-s001.zip › Figure S1.pdf]

Figure S2. Information on amino acid standards.

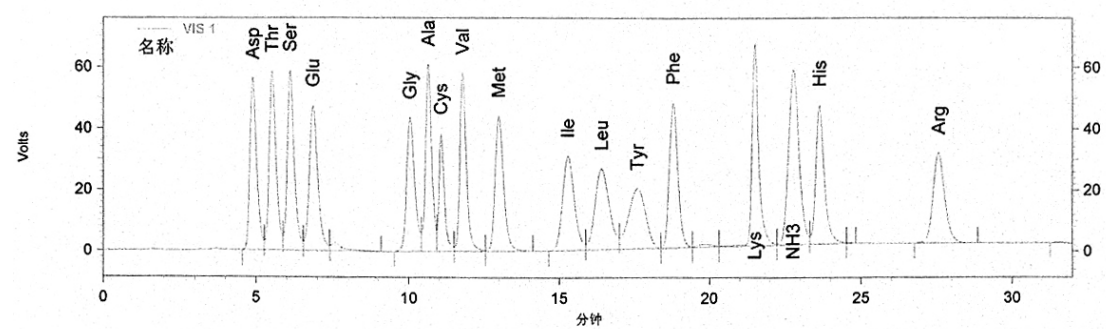

Supplement: Supplementary file 1 [file foods-14-03175-s001.zip › Figure S2.pdf]
